# Comparative Tandem Mass Tag-Based Quantitative Proteomic Analysis of *Tachaea chinensis* Isopod During Parasitism

**DOI:** 10.3389/fcimb.2019.00350

**Published:** 2019-10-11

**Authors:** Yingdong Li, Xin Li, Zhibin Han, Weibin Xu, Xiaodong Li, Qijun Chen

**Affiliations:** College of Animal Science and Veterinary Medicine, Shenyang Agricultural University, Shenyang, China

**Keywords:** comparative proteomic, isopod parasite, *Tachaea chinensis*, blood-sucking, during parasitism

## Abstract

Parasitic isopods perforate and attach to the host integument via the mandibles and then feed on hemolymph and exudate from the wounds. Such isopods attack a variety of commercially important fish and crustacean hosts. Similar to other hematophagous parasites, isopods may also employ biomolecules that affect host blood conglutination and defense systems. In the present study, a tandem mass tag-based quantitative proteomic approach was used to identify differentially expressed proteins in *Tachaea chinensis* parasites of shrimp, by comparing parasitic (fed) and pre-parasitic (unfed) individuals. We identified 888 proteins from a total of 1,510 peptides, with a significant difference in 129 between the fed and unfed groups. Among these, 37 were upregulated and 92 were downregulated in unfed *T. chinensis*. This indicates that *T. chinensis* may require more energy before parasitism during its search for a host. In addition, as is the case for other blood-sucking parasites, it might secrete antihemostatic, anti-inflammatory, and immunomodulatory molecules to facilitate blood meal acquisition. To our knowledge, this study is the first to use a TMT-based proteomic approach to analyze the proteome of isopod parasites, and the results will facilitate our understanding of the molecular mechanisms of isopod parasitism on crustaceans.

## Introduction

Hematophagous parasites such as ticks, mosquitoes, fleas, and leeches can bypass the clotting system of their vertebrate hosts by secreting anticoagulant compounds (Shu et al., 2010; Santiago et al., 2017). Isopod parasites mainly feed on blood (hemolymph) from hosts after perforating the integument with their mandibles, which can cause stress, tissue damage, secondary infection, and mortality (Wilson, 2008; Poore and Bruce, 2012; Williams and Boyko, 2012; Smit et al., 2014). There is evidence that isopod parasites may also inject anticoagulants or other compounds directly into the host so as to evade defensive responses (Nair and Nair, 1983; Manship et al., 2012). At present, only a few proteins for this purpose have been identified, including trypsin inhibitors and anticoagulants in *Paragnathia formica*. Identification of isopod anticoagulant agents may be useful in fully understanding parasite modulation of the host physiological system. This information may be valuable for the identification of novel target antigens and antithrombotic drug development.

*Tachaea chinensis*, one of the most common ectoparasites of farm-raised shrimps, is widely distributed in China and neighboring countries (Lu et al., 2007; Hua et al., 2018; Li et al., 2018). It is normally approximately 0.7 cm in size and can easily be observed with the naked eye following parasitization. After attacking its host, this isopod invariably remains attached until either the shrimp dies or another shrimp comes within close proximity. Over the past 2 years, *T. chinensis* has been shown to parasitize over 80% of aquacultured *Palaemonetes sinensis* (Li et al., 2018) and 90% of *Exopalaemon carinicauda* (Li et al., 2019). Additional research on isopod parasitization mechanisms is necessary to develop effective methods of control for use in shrimp aquaculture.

We performed tandem mass tag (TMT)-based quantitative proteomic profiling of *T. chinensis* to compare protein expression in fed and unfed parasites and gain insight into their integrated molecular mechanisms and host responses. This will provide an empirical basis for disease prevention and control efforts and support further research on the molecular biology of isopods.

## Materials and Methods

### Ethics Statement

Our study did not involve endangered or protected species. In China, the capture of isopod parasites and their host shrimp from rice fields does not require specific permits. All efforts were made to minimize animal suffering and discomfort. The experimental protocol was approved by the Animal Ethics Committee of Shenyang Agriculture University.

### Animals

*Tachaea chinensis* isopod parasites (0.82 ± 0.17 cm) and their host shrimp *P. sinensis* (3.48 ± 0.35 g) used in this study were collected from a rice field in Panjin City, Liaoning Province, China, in November 2018, and transported to the aquaculture laboratory at Shenyang Agricultural University. They were acclimated in two 300 L^2^, fiberglass recirculation tanks with a circular flow system. Water temperature was maintained at 24 ± 0.5°C, and the photoperiod was set at a 12:12 h light:dark cycle. After 2 weeks of acclimatization, 20 healthy *T. chinensis* were transferred to individual plastic tanks (15.8 cm diameter and 32.1 cm height), each containing 5 L water, with the same environmental conditions as during the acclimatization period.

Following this, one *P. sinensis* each was placed in 10 of the prepared tanks, forming the fed group, with the other tanks kept as unfed pre-parasitism controls. After 7 d, fed and unfed *T. chinensis* were removed to individual 2 mL RNAse-free tubes and immediately frozen in liquid nitrogen for storage until protein extraction.

### Protein Extraction and SDS-PAGE Analysis

Three fed and three unfed isopods were ground into powder and vortexed in 600 μL SDT buffer (pH 8.0, 4% SDS, 150 mM Tris-HCl, 1 mM DTT), respectively. The mixtures were heated at 100°C for 10 min, then sonicated at 35 W for 4 s, with 7 s intervals, for 10 min. These were centrifuged at 14,000 × *g* for 30 min, and supernatants were collected into 0.22 μm filter tubes. One microliter of the underlayer liquid of each sample was used for BCA quantitative analysis, and 1 μg of the protein sample from each group was subjected to SDS-PAGE (12.5% resolving gels) analysis (Figure S1).

### Protein Digestion and TMT Labeling

Briefly, six protein concentrates (300 μg each) were mixed in ultrafiltration filtrate tubes (30 kDa cut-off, Sartorius, Gottingen, Germany) with 200 μL urea buffer (8 M urea, 150 mM Tris-HCl, pH 8.0), and the sample was centrifuged at 14,000 × *g* at 20°C for 30 min. The sample was washed twice by adding 200 μL UA and centrifuged at 14,000 × *g* at 20°C for 30 min. The flow-through from the collection tube was discarded. Next, 100 μL of indole-3-acetic acid (IAA) solution (50 mM IAA in UA buffer) was added to the filter tube and vortexed at 600 rpm in a Thermomixer comfort incubator (Eppendorf, Germany) for 1 min. Subsequently, the sample was incubated at room temperature for 30 min in the dark and centrifuged at 14,000 × *g* for 30 min at 20°C. Next, 100 μL UA was added to the filter unit, which was centrifuged at 14,000 × *g* for 20 min; this was carried out three times. The protein suspension in the filtrate tube was subjected to enzyme digestion with 52 μL of trypsin (Promega, Madison, WI, USA) buffer [6 μg trypsin (0.5 μg/μL) in 40 μL of dissolution buffer] for 16–18 h at 37°C. Finally, the filter unit was transferred to a new tube and centrifuged at 14,000 × *g* for 30 min. Peptides were collected in the filtrate and the peptide concentration was measured based on the optical density at 280 nm (OD280).

TMT labeling was performed using the TMT6plex™ Isobaric Label Reagent Set (Thermo Scientific) according to the manufacturer's instructions. The proteins in the unfed group were labeled with reagents 119, 127, and 128, and those in the infected group were labeled with reagents 129, 130, and 131. The labeling solution reaction was then incubated at room temperature for 1.5 h prior to further analysis.

### SCX Fractionation and LC-MS/MS Analysis

SCX fractionation and LC-MS/MS analysis were conducted according to a published protocol (Xiao et al., 2019). The TMT-labeled samples were analyzed using an Easy-nLC nanoflow HPLC system connected to Orbitrap-Elite (Thermo Fisher Scientific, San Jose, CA, USA). One microgram of each sample was loaded onto each of two Thermo Scientific EASY columns using an autosampler at a flow rate of 200 nL/min. The sequential separation of peptides on Thermo Scientific EASY trap column (100 μm × 2 cm, 5 μm, 100 Å, C18) and analytical column (75 μm × 25 cm, 5 μm, 100 Å, C18) was achieved using a segmented 1 h gradient from 5 to 28% Solvent B (0.1% formic acid in 100% ACN) for 40 min, followed by 28–90% Solvent B for 2 min, and 90% Solvent B for 18 min. The column was re-equilibrated to its initial highly aqueous solvent composition before each analysis. The mass spectrometer was operated in positive ion mode, and MS spectra were acquired over a range of 350–2,000 m/z. The resolving powers of the MS scan and MS/MS scan at 100 m/z for the Orbitrap Elite were set as 60,000 and 15,000, respectively. The top 16 most intense signals in the acquired MS spectra were selected for further MS/MS analysis. The isolation window was 2 m/z, and ions were fragmented through high energy collisional dissociation with normalized collision energies of 35 eV. The maximum ion injection times were set at 10 ms for the survey scan and 100 ms for the MS/MS scans, and the automatic gain control target values for full scan modes was set to 1 e 6 and for MS/MS was 5 e 4. The dynamic exclusion duration was 30 s.

### Data Analysis

Raw files were analyzed using the Proteome Discoverer 2.1 software (Thermo Fisher Scientific). A search for the fragmentation spectra was performed using the MASCOT search engine embedded in Proteome Discoverer against the NCBI_Peracarida_91190_20190313.fasta database. The following search parameters were used: monoisotopic mass, trypsin as the cleavage enzyme, two missed cleavages, TMT labeling, and carbamidomethylating of cysteine as fixed modifications. Peptide charges of 2^+^, 3^+^, and 4^+^ and the oxidation of methionine were specified as variable modifications. The mass tolerance was set to 20 ppm for precursor ions and to 0.1 Da for fragment ions. The results were filtered based on a false discovery rate of no more than 1%. The relative quantitative analysis of the proteins in the samples based on the ratios of TMT reporter ions from all unique peptides representing each protein was performed using Proteome Discoverer (version 2.1). The relative peak intensities of the TMT reporter ions released in each of the MS/MS spectra were used. Then, the final ratios obtained from the relative protein quantifications were normalized based on the median average protein quantification ratio. The fold change was set to >1.2 for protein upregulation and <0.85 for protein downregulation (*P* < 0.05). Protein functional annotation was conducted using the Universal Protein (UniProt) database. Gene Ontology (GO) and Kyoto Encyclopedia of Genes and Genomes (KEGG) databases were also used.

The mass spectrometry proteomics data have been deposited in the ProteomeXchange Consortium via the PRIDE partner repository (identifier PXD015247). All analyzed data are available from the corresponding author upon reasonable request.

### Verification of Protein Quantifications Using PRM Analysis

Parallel reaction monitoring (PRM) was used to verify the TMT-based quantitative proteomics results. Briefly, 2 μg of peptide from each sample was taken for LC-PRM/MS analysis. After sample loading, chromatographic separation was performed using a Thermo Scientific EASY-nLC nano-HPLC system, with two buffers. Solution A was 0.1% formic acid aqueous solution and solution B was a mixed solution of 0.1% formic acid, 95% acetonitrile, and water. The column was first equilibrated with 95% solution A. The sample was injected into a Trap column (100 μm × 20 mm, 5 μm C18, Dr. Maisch GmbH) and subjected to gradient separation through a chromatography column (75 μm × 150 mm, 3 μm C18, Dr. Maisch GmbH) at a flow rate of 250 nL/min. The liquid phase separation gradient was as follows: 0–25 min, linear gradient of B liquid from 5 to 18%; 25–45 min, linear gradient of B liquid from 18 to 50%; 45–48 min, linear gradient of B liquid from 50 to 95%; and 48–60 min, B liquid maintained at 95%. The peptides were separated and subjected to targeted PRM/MS using a Q-Exactive mass spectrometer (Thermo Scientific) for 60 min. The parameters were set as follows: detection mode, positive mode; parent ion scanning range, 350–1,500 m/z; capillary voltage, 1.8 kV; isolation width, 1.6 Th; first-order MS resolution, 70,000 at m/z 200; AGC target, 3 e 6; first-level maximum IT, 250 ms. Peptide secondary MS were obtained as follows: for each full scan, target peptides of precursor m/z were sequentially selected based on the inclusion list for second-order MS (MS2) scan. The parameters used were as follows: resolution, 35,000 at m/z 200; AGC target, 3 e 6; Level 2 maximum IT, 120 ms; MS2 activation type, HCD; peptide fragmentation, nitrogen; isolation window, 2.0 Th; normalized collision energy, 28 eV. Four proteins were randomly selected from the global proteomics analysis (re-labeled peptide GISNEGQNASIK^*^ as the reference standard mixture), including phosphoglucomutase (TFTTQETITNAATSAK), glucose-6-phosphate isomerase (LGAENFVFFHPR), xylose isomerase (YFGNLMDAGR, LSICGEESFGTGSDHIR), and malate dehydrogenase (IFGVTTLDIVR, IQDAGTEVVK). Skyline 3.5 was used to generate an initial PRM transition pair list for the four candidate DEPs.

## Results

### SDS-PAGE Analysis and Protein Profiling

**Figure 1:**
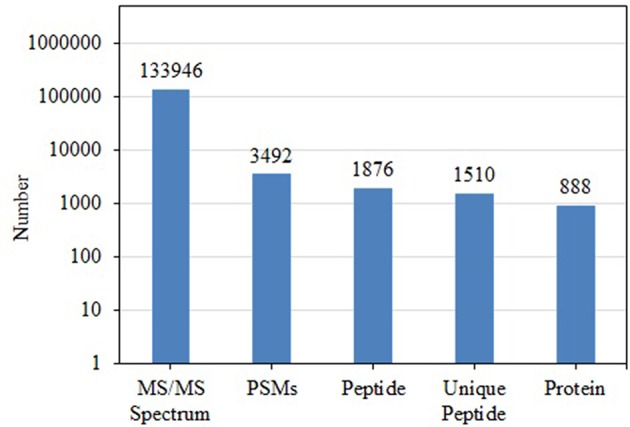
Total numbers of tandem mass spectrometry spectra (MS/MS spectrum), peptide-spectrum matches (PSMs), peptides, unique peptides, and proteins in *Tachaea chinensis*.

**Figure 2:**
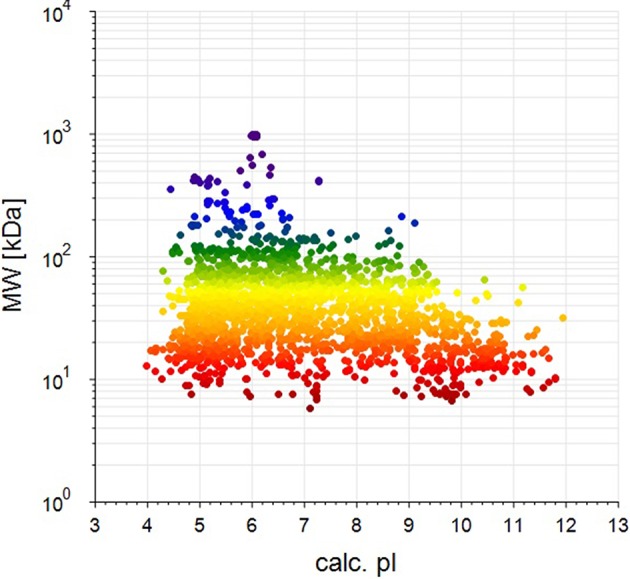
Distribution of identified proteins with different molecular weights (the weight and pI values are for the target proteins from the NCBI_Peracarida_91190_20190313.fasta database).

BCA results indicated protein concentrations of 15.60, 20.94, and 21.55 μg/μL in fed isopods and 19.06, 25.07, and 22.03 μg/μL in unfed isopods. TMT analysis indicated 3,492 queries in the 133,946 spectra. Among them, a total of 888 unique proteins were identified across 1,510 peptides (Figure 1). There were 139 proteins between 0 and 20 kDa, and 282, 241, 92, 45, and 89 proteins of 20–40, 40–60, 60–80, and 80–100 kDa, respectively. Eighty-nine proteins had a mass of over 100 kDa (Figure 2).

### Differentially Expressed Protein Analysis

**Figure 3:**
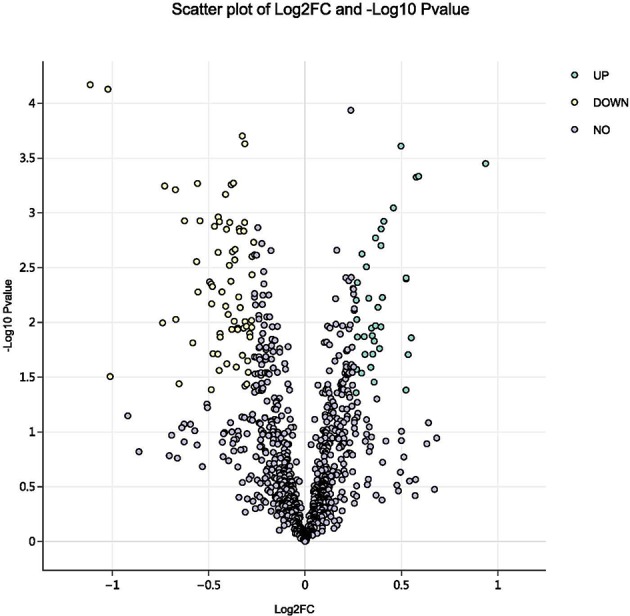
Volcano plot of all proteins identified in the proteome database, for the fed and unfed parasite groups. Yellow points: differentially expressed proteins that were significantly downregulated after parasitism (fold change > 1.2; *p* < 0.05). Green points: differentially expressed proteins that were significantly upregulated after parasitism (fold change < 0.8; *p* < 0.05).

Using a 1.2-fold increase or decrease in protein expression as a benchmark for a physiologically significant change, 129 differentially expressed proteins (DEPs, *p* < 0.05) were identified between the control and parasitism groups (Figure 3). Within these DEPs, 37 were upregulated and 92 were downregulated (Table S1, Figure S2) before parasitism.

**Figure 4:**
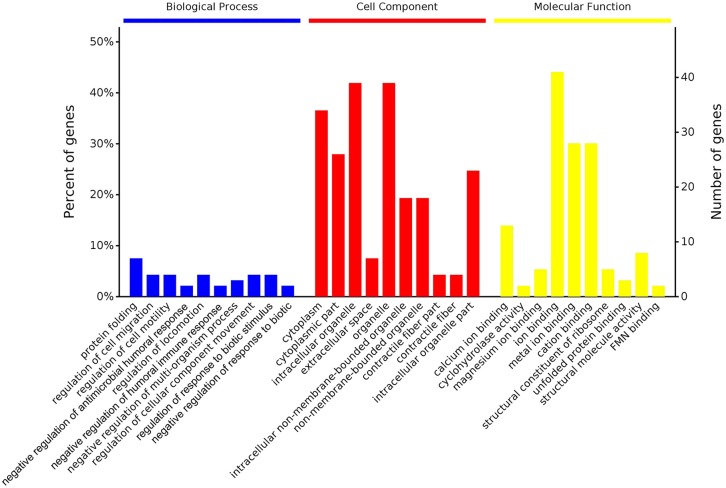
GO functional classification of the identified proteins of the top 10 pathways identified in the proteome database, for the fed and unfed parasite groups, in the biological process, cell component, and molecular function categories.

**Figure 5:**
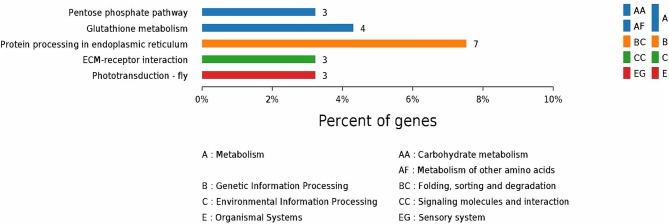
KEGG annotation of the top five pathways from the proteome database of the fed and unfed parasite groups.

**Figure 6:**
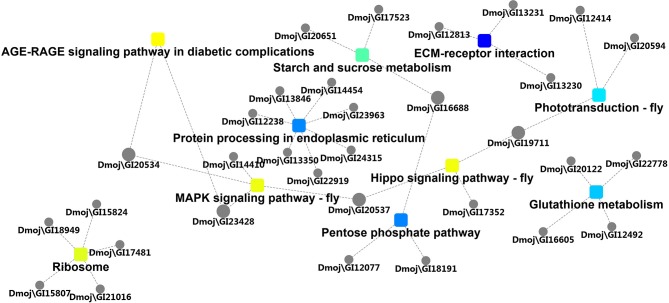
Protein-protein interaction network analysis of the three major network groups.

According to GO enrichment analysis, 1,256, 206, and 274 proteins were enriched for the categories biological process, cell component, and molecular function, respectively, and 178, 71, and 83 of these were statistically significant (Figure S2). The top 10 significant pathways are shown in Figure 4. Fifty-seven KEGG proteins were enriched, and five were statistically significant. Their functions included ECM-receptor interaction, pentose phosphate pathways, protein processing in the endoplasmic reticulum, glutathione metabolism, and phototransduction-fly, with 3, 3, 7, 4, and 3 proteins mapped to them, respectively (Figure 5). According to the protein–protein interaction network analysis, the three major clusters of interaction were protein processing in endoplasmic reticulum, ribosomes, and glutathione metabolism (Figure 6).

### Candidate Proteins Related to Energy and Digestive Metabolism

Some differentially expressed proteins related to energy metabolism were significantly upregulated in unfed *T. chinensis*, including calcium-transporting ATPase sarcoplasmic/endoplasmic reticulum type-like proteins, NADH dehydrogenase [ubiquinone] flavoprotein 1, mitochondrial-like Na^+^/K^+^-ATPase, partial sodium potassium ATPase alpha subunit, partial smooth endoplasmic reticulum calcium ATPase, NADH dehydrogenase subunit 1 (mitochondrion), and cyclic AMP-responsive element-binding protein 1 (Table 1). Among the 92 significantly upregulated differentially expressed proteins, 20 were related to digestive metabolism, including 7, 11, and 2 in carbohydrate, protein, and lipid metabolism, respectively (Table 1).

**Table 1:** Differentially expressed proteins involved in energy and digestive metabolism, comparing the fed and unfed *Tachaea chinensis*.

**Accession**	**Description**	**Coverage**	**Peptides**	**PSMs**	**AAs**	**MW (kDa)**	**Calc. pI**	**FC Unfed/Fed**	***P*-value**
**ENERGY METABOLISM**									
1371969814	Calcium-transporting ATPase sarcoplasmic/endoplasmic reticulum type-like	13.89	13	36	1,001	109.50	5.52	1.20	0.04
1067084430	NADH dehydrogenase flavoprotein 1, mitochondrial-like	8.55	3	3	468	50.90	8.21	1.20	0.01
1547583175	Na^+^/K^+^-ATPase, partial	29.70	5	9	202	22.40	6.77	1.28	0.02
1246309936	Sodium potassium ATPase alpha subunit, partial	25.99	5	9	227	25.20	5.39	1.28	0.01
26324131	Smooth endoplasmic reticulum calcium ATPase	18.96	17	46	1,002	109.70	5.36	1.32	0.01
1220106012	NADH dehydrogenase subunit 1 (mitochondrion)	3.56	1	1	309	34.70	8.88	1.41	0.00
1561970059	Cyclic AMP-responsive element-binding protein 1	1.98	1	1	353	37.50	7.56	1.44	0.00
**DIGESTIVE METABOLISM**									
**Carbohydrate Metabolism**									
1371969117	Phosphoglucomutase	3.02	1	1	563	61.40	5.26	0.68	0.00
1562001515	Trehalase	1.46	1	1	548	62.80	5.26	0.73	0.03
262305275	Glycogen synthase, partial	6.31	2	3	317	36.20	7.64	0.85	0.01
1067085998	UDP-glucuronosyltransferase 2B14-like	3.85	1	5	156	17.80	9.57	0.76	0.00
1371968467	Fructose 1,6-bisphosphatase	8.33	2	3	276	30.00	5.35	0.78	0.01
1371966397	Kynurenine–oxoglutarate transaminase 3-like, partial	2.69	1	2	484	53.90	7.71	0.83	0.00
1562032021	Ubiquitin carboxyl-terminal hydrolase 7	1.18	1	1	1,267	145.50	5.77	0.83	0.04
**Protein Metabolism**									
1067064770	Aminopeptidase N-like	0.59	1	2	1,851	208.50	4.91	0.63	0.00
1371969475	26S protease regulatory subunit 6B	5.56	3	3	414	46.70	5.58	0.65	0.00
1562025605	Protein disulfide-isomerase A6	2.27	1	1	441	48.30	5.57	0.68	0.01
1371970147	Nucleoside diphosphate kinase	7.73	1	2	181	20.20	8.40	0.74	0.01
1562030658	Protein disulfide-isomerase	5.17	3	4	522	58.70	4.77	0.75	0.00
1371969013	Dolichyl-diphosphooligosaccharide–protein glycosyltransferase subunit STT3A-like	1.66	1	1	722	82.10	7.87	0.78	0.01
1562029772	Ubiquitin-protein ligase E3C	0.87	1	2	1,032	117.50	6.76	0.78	0.01
1371965865	Serine/threonine-protein phosphatase 4 regulatory subunit 1-like	0.52	1	1	1,340	149.30	4.65	0.80	0.00
1562011562	Peptidyl-prolyl cis-trans isomerase FKBP7	13.81	4	7	210	23.50	4.79	0.80	0.00
1562027280	Proteasome subunit beta type-5	4.61	1	2	282	31.50	6.65	0.85	0.02
1561992339	Putative serine/threonine-protein phosphatase PP2A regulatory subunit	10.44	4	5	479	53.00	5.21	0.85	0.02
**Lipid Metabolism**									
1562029772	Ubiquitin-protein ligase E3C	0.87	1	2	1,032	117.50	6.76	0.78	0.01
1562030180	Fatty acid synthase	0.57	1	2	2,263	248.70	5.49	0.81	0.02

### Candidate Proteins Related to Blood Sucking

Three hemocyanin proteins showed the greatest difference in expression between fed and unfed isopods (Table S1). Differentially expressed proteins related to blood sucking, including inorganic pyrophosphatase-like isoform X1, alpha-2-macroglobulin, bestrophin homolog, calreticulin, barrier-to-autointegration factor putative salivary alkaline phosphatase, inorganic pyrophosphatase-like isoform X1, neurocalcin homolog isoform X3, and insulin receptor substrate 2-B were significantly upregulated after parasitism (Table 2). Four heat shock proteins and two stress-activated proteins were also upregulated.

**Table 2:** Differentially expressed proteins involved in blood sucking, comparing the fed and unfed *Tachaea chinensis*.

**Accession**	**Description**	**Coverage**	**Peptides**	**PSMs**	**AAs**	**MW (kDa)**	**Calc. pI**	**FC Fed/Unfed**	***P*-value**
**BLOOD SUCKING**									
427782987	Putative salivary alkaline phosphatase	1.68	1	1	1	1.0	535.00	0.45	6.60
1561973869	Barrier-to-autointegration factor	11.11	1	1	90	10.1	5.90	0.67	0.02
1067116995	Inorganic pyrophosphatase-like isoform X1	3.41	1	1	410	46.2	5.08	0.72	0.00
1067085871	Calcineurin subunit B type 2 isoform X1	4.69	1	1	213	23.5	4.97	0.75	0.02
1067070998	Neurocalcin homolog isoform X3	10.53	2	2	190	21.9	5.25	0.74	0.01
164632859	Alpha-2-macroglobulin, partial	3.85	2	3	571	61.7	4.94	0.75	0.00
1562026595	Calreticulin, partial	13.03	3	5	330	38.4	4.93	0.81	0.00
1202288454	Insulin receptor substrate 2-B, partial	2.35	1	1	426	46.2	6.38	0.82	0.01
1067101392	Mitogen-activated protein kinase 14B-like	4.08	1	1	392	44.0	7.30	0.82	0.03
1562031882	PI-actitoxin-Aeq3a	6.51	1	1	169	19.5	4.81	0.83	0.02
1562036618	Bleomycin hydrolase	4.76	1	1	252	29.0	6.64	0.85	0.04
1371956987	Peroxiredoxin, partial	9.65	1	1	114	12.4	5.48	1.20	0.03
1371959777	Galactose ABC transporter substrate-binding protein	3.26	1	1	337	37.1	5.03	1.50	0.00
**HEAT SHOCK PROTEIN**									
190014500	70 kDa heat shock protein	7.64	4	10	641	70.1	5.53	0.72	0.00
1561987640	Endoplasmin	3.99	3	5	803	92.1	5.02	0.77	0.00
1561990168	60 kDa heat shock protein, mitochondrial	14.19	8	13	571	60.5	5.41	0.81	0.01
1371970216	Heat shock protein HSP 90-alpha-like	7.49	5	7	708	82.0	5.05	0.83	0.00
1371965305	Stress-activated protein kinase JNK-like	3.14	1	1	509	57.2	7.40	0.83	0.04
1562029713	Putative heat shock protein HSP 90-alpha A4	10.86	5	8	488	55.7	5.08	0.83	0.00

### PRM Results

**Figure 7:**
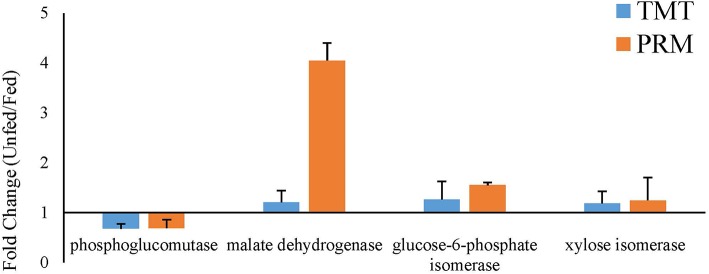
Verification of the expression profiles of randomly selected significantly changed proteins comparing the fed and unfed *T. chinensis* groups, as determined by parallel reaction monitoring (PRM) and tandem mass tag (TMT) analysis. The data on the relative expression of proteins, for use in the TMT analysis, were obtained from Tables 1, 2. The data on the relative expression of proteins for use in the PRM analysis were calculated relative to the expression of the relabeled peptide GISNEGQNASIK*.

To validate the proteomic data, three proteins that were upregulated after parasitism (phosphoglucomutase, glucose-6-phosphate isomerase, and xylose isomerase) and one that was downregulated after parasitism (malate dehydrogenase) were selected for PRM analysis. In both the fed and unfed groups, the validated proteins showed expression trends similar to the proteomic expression trends, suggesting that the proteomic data were reliable (Figure 7).

## Discussion

Hematophagous parasites use antihemostatic, anti-inflammatory, and immunomodulatory compounds to facilitate blood meal acquisition during feeding (Shu et al., 2010; Hajdusek et al., 2013; Eliane et al., 2017; Santiago et al., 2017; Jalovecka et al., 2018). These promote the survival and establishment of pathogens in the host (Boonsriroj et al., 2015; Eliane et al., 2017). Identification and characterization of such compounds can elucidate molecular mechanisms of interaction among parasites, pathogens, and hosts, revealing new vaccine targets (Mcgowan et al., 1982; Slovák et al., 2013; Oleaga et al., 2015; Yu et al., 2015; Eliane et al., 2017; Tirloni et al., 2017). Although many researchers have assumed that isopod parasites are hematophagous parasites and feed mainly on fish blood or crustacean hemolymph, no prior studies have demonstrated this directly. Moreover, in contrast to ticks and mosquitoes, there is a lack of comprehensive genomic resources for isopod parasites to investigate infection and response mechanisms. We performed, to our knowledge, the first comparative proteomic analysis between fed and unfed *T. chinensis* to gain a better understanding of its feeding and antigen-related proteins. However, because very few protein sequences for isopods are available in the NCBI protein database and all proteins identified were based on homologous proteins, we were able to identify only 888 proteins. Moreover, to avoid potential contamination of the host protein samples and to screen out proteins associated with parasitic processing, we also searched the NCBI protein database for proteins of Peracarida and blood-sucking insects (ticks and mosquitos); we found that proteins from *Armadillidium vulgare* (442, 51.76%), *Hyalella azteca* (227, 26.58%), and *Hirondellea gigas* (143, 16.74%) were similar to those present in our samples (Table S1).

Comparative proteomic analysis of unfed and fed *T. chinensis* indicated 129 proteins expressed significantly, with 37 upregulated and 92 downregulated before parasitism. Among the 37 upregulated proteins, seven were identified as contributing to ATP synthesis (Table 1). These results suggest that high energy metabolic proteins are expressed in pre-parasitism isopods while searching for hosts. Meanwhile, previous research has shown that genes related to ATP generation in host shrimp *P. sinensis* are upregulated under parasitism, which might be induced by a rapid depletion of ATP content in the parasite itself after parasitism, during its development and reproduction (Li et al., 2018). A similar profile has been demonstrated in fleas, where energy costs for feeding are lower than those in unfed individuals (Sarfati, 2005). Moreover, in crustaceans, because proteins are major contributors to hemolymph density (Simonetta et al., 2011), in their parasites, the major digestive enzymes are thought to be proteases. Eleven proteolytic enzymes were identified among the 129 DEPs, and all were upregulated after parasitism (Table 1). Seven carbohydrate metabolism-related proteins were also upregulated, suggesting that hemolymph glucose may also be an important nutrient for *T. chinensis*. Trehalose, which regulates energy metabolism and glucose generation via trehalose catabolism, was also upregulated after parasitism, suggesting that access to host trehalose is an important feature of isopod parasites.

Many researchers have assumed that isopod parasites can feed on fish blood or crustacean hemolymph, comparable to an “aquatic mosquito” (Wilson, 2008; Manship et al., 2012; Smit et al., 2014; Nagler et al., 2017). We previously found that hemocyanin in *P. sinensis* is significantly downregulated following parasitism by *T. chinensis* (Li et al., 2019). The present results indicate that hemocyanin in *T. chinensis* was significantly upregulated after parasitism, suggesting a role in obtaining hemolymph from *P. sinensis*. In hematophagous arthropods such as ticks, mosquitos, fleas, and flies, hematophagy evolved independently over millions of years, leading to various morphological adaptations and diverse strategies to overcome the barriers imposed by hosts (Ribeiro, 1995; Garcia et al., 2015). However, one example of convergent evolution and adaptation for hematophagy is the development of highly functional salivary glands, which produce a large diversity of anticlotting, antiplatelet, vasodilatory, and pathogen-transporting substances (Kim et al., 2016; Tirloni et al., 2016). Although it is known that isopod parasite morphology has been strongly modified for parasitism over 168 million years (Nagler et al., 2017), the type and composition of blood-sucking-related substances remain to be understood.

During blood sucking, the salivary gland of hematophagous parasites produces a cocktail of anti-hemostatic, anti-inflammatory, and immunomodulatory molecules that facilitate blood-meal acquisition. Saravanan et al. (2003) found that α_2_-macroglobulin, which they consider to have an anticoagulant function, is highly expressed in tick salivary glands. In the present study, α_2_-macroglobulin was significantly upregulated in isopods that had recently fed, which indicates that *T. chinensis* may also secrete anticoagulatory molecules during feeding. Moreover, Saurabh et al. (2011) found that fish macroglobulin was downregulated during *Argulus siamensis* parasitism. Similarly, we previously found that shrimp macroglobulin was downregulated during *T. chinensis* parasitism. Moreover, calreticulin, which functions in hematophagy in ticks through host immunosuppression or antihemostasis (Parizi et al., 2009), was also significantly upregulated after parasitism in the present study. Furthermore, calcineurin subunit B type 2 isoform X1, putative salivary alkaline phosphatase, insulin receptor substrate 2-B, PI-actitoxin-Aeq3a, and neurocalcin homolog isoform X3, which are similar to substances in the salivary glands of hematophagous parasites, were also upregulated in *T. chinensis* during feeding (Kim et al., 2015). However, the interaction of those proteins between parasites and hosts and the mechanisms of paralyzation and inhibition of inflammation remain unclear and require further study.

Anticlotting-related proteins are considered potential targets for the development of new drugs and vaccines to control and prevent ectoparasites such as ticks (Oleaga et al., 2017), mosquitos (Popova-Butler and Donald, 2009; Tetreau et al., 2012), *Schistosoma* (Delcroix et al., 2006), and sea louse (Carpio et al., 2011, 2016). However, the hosts of these parasites are vertebrates, in which the composition of the coagulation system differs from that of invertebrates. In shrimps, the hemolymph clotting system comprises transglutaminase and clotting proteins and plays an important role in the innate immune response and prevention of blood loss during injury and wound healing (Maningas et al., 2013). Isopod parasites may inject anticoagulants or other compounds directly into the blood to obtain a blood meal (Nair and Nair, 1983; Manship et al., 2012), but the constituents of these compounds are still unclear. Here, we identified two protein disulfide-isomerase (PDI) proteins that participate in parasite–host cell interactions. PDI-specific antibodies may constitute part of the mucosal antibody repertoire, which is possibly involved in defense against parasites. The potential roles of those proteins for isopod parasite control will be investigated in our further study.

In conclusion, our results represent the first comparative proteomic study to detect key proteins from the whole body of fed and unfed *T. chinensis* isopod parasites. Owing to the lack of genomic data, we were able to identify only 888 proteins, and all were found in both stages. Differentially expressed proteins related to energy metabolism were upregulated in unfed individuals, particularly those involved in ATP generation. This may indicate that *T. chinensis* may require considerable energy during its search for a host. We also found that similar to other hematophagous parasites, *T. chinensis* secretes antihemostatic, anti-inflammatory, and immunomodulatory molecules to facilitate blood meal acquisition. Our study provides valuable empirical data that will support future molecular research on isopod parasitization of crustaceans.

## Data Availability Statement

The raw data supporting the conclusions of this manuscript will be made available by the authors, without undue reservation, to any qualified researcher.

## Author Contributions

YL and QC designed the experiments. XinL and ZH generated biological samples. XinL, ZH, and WX performed the experiments. YL, XinL, and QC analyzed data. YL and WX performed statistic data analysis. YL and XiaL contributed reagents, materials and analysis tools. YL, XinL, ZH, and WX wrote the paper. All authors read and approved the final manuscript.

### Conflict of Interest

The authors declare that the research was conducted in the absence of any commercial or financial relationships that could be construed as a potential conflict of interest.

## Supplementary Material

Figure S1 Separation of proteins by SDS-PAGE from six unfed (A1, A2, and A3) and fed (B1, B2, and B3) *Tachaea chinensis*.

Figure S2 Hierarchical clustering analysis of differentially expressed proteins in unfed (A1, A2, and A3) and fed (B1, B2, and B3) *Tachaea chinensis*.

Table S1 Lists of proteins indented from *Tachaea chinensis*.
