# Supplementary figures and images for: Comparative Tandem Mass Tag-Based Quantitative Proteomic Analysis of Tachaea chinensis Isopod During Parasitism
Source: Front Cell Infect Microbiol. 2019 Oct 11;9:350. doi: 10.3389/fcimb.2019.00350 (PMC6798089; doi:10.3389/fcimb.2019.00350)

MW/KDa

116

66.2

45

35

25

18.4

14.4

M

A1

A2

A3

B1

B2

B3

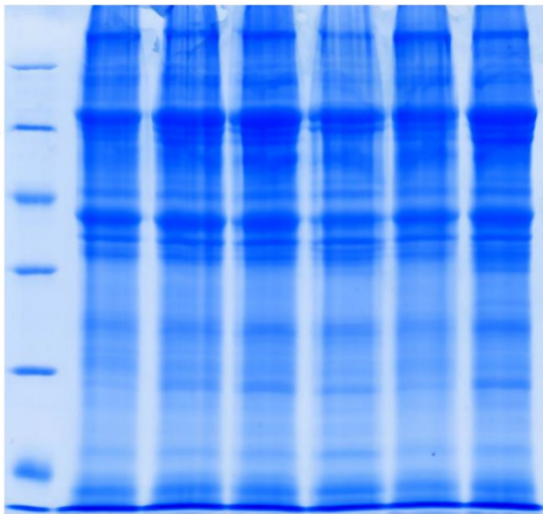

Supplement: Figure S1 — Separation of proteins by SDS-PAGE from six unfed (A1, A2, and A3) and fed (B1, B2, and B3) Tachaea chinensis. [file Image_1.PDF]

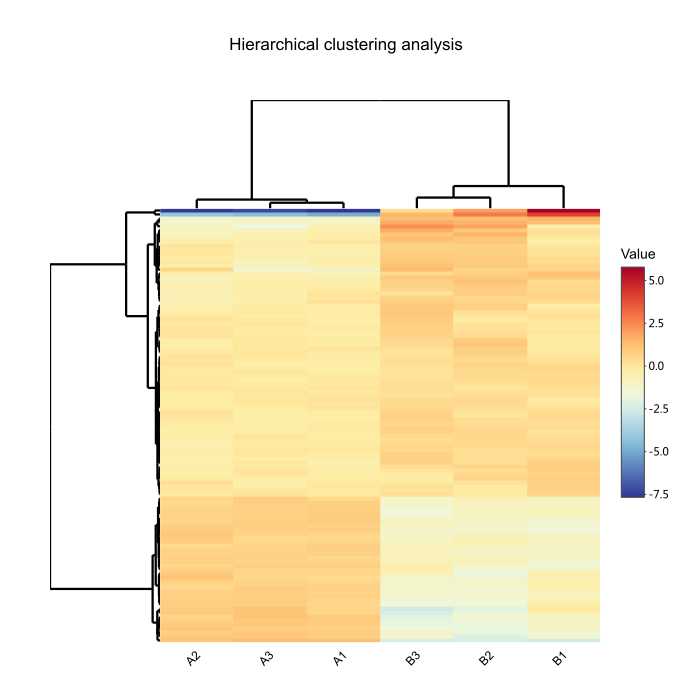

Supplement: Figure S2 — Hierarchical clustering analysis of differentially expressed proteins in unfed (A1, A2, and A3) and fed (B1, B2, and B3) Tachaea chinensis. [file Image_2.PNG]
